# Dalbavancin to facilitate early discharge in the treatment of complex musculoskeletal infections: a multi-centre real-life application

**DOI:** 10.5194/jbji-10-93-2025

**Published:** 2025-03-31

**Authors:** Tariq Azamgarhi, Simon Warren, Antonia Scobie, Natasha Karunaharan, Cristina Perez-Sanchez, Rebecca Houghton, Salma Hassan, Julie Lourtet-Hascoët, Hannah Kershaw, Parham Sendi, Kordo Saeed

**Affiliations:** 1 Pharmacy Department, Royal National Orthopaedic Hospital NHS Trust, Brockley Hill, Stanmore, HA7 4LP, United Kingdom; 2 Bone Infection Unit, Royal National Orthopaedic Hospital NHS Trust, Brockley Hill, Stanmore, HA7 4LP, United Kingdom; 3 Pharmacy Department, University Hospital Southampton NHS Foundation Trust, Southampton, United Kingdom; 4 Department of Microbiology, Hampshire Hospitals NHS Foundation Trust, Hampshire, Basingstoke, United Kingdom; 5 Infectious Diseases and Microbiology Department, Hôpital Joseph Ducuing, Toulouse, France; 6 Clinical Microbiology Department, Hôpital Saint Joseph, Paris, France; 7 Pharmacy Department, Royal Free Hospital NHS Foundation Trust, London, United Kingdom; 8 Institute for Infectious Diseases, University of Bern, Bern, Switzerland; 9 Department of Infection, University Hospital Southampton NHS Foundation Trust, Southampton, United Kingdom; 10 Clinical and Experimental Sciences, University of Southampton, Southampton, United Kingdom

## Abstract

Dalbavancin is a lipoglycopeptide with a half-life of 14 d, significantly reducing the need for daily antibiotic dosing. Although dalbavancin is approved for acute bacterial skin and skin structure infections, its off-label use in complex musculoskeletal infection (MSKI) is increasing. Evidence on its effectiveness for MSKI, especially in facilitating early discharge for patients unsuitable for oral or OPAT (outpatient parenteral antimicrobial therapy) treatments, is limited.

This multi-centre observational study aims to evaluate dalbavancin's role in facilitating discharge and improving clinical outcomes in MSKI. **Method**: this study included adult patients treated with dalbavancin between January 2017 and December 2022 across five hospitals in the UK and France. Data on patient demographics, clinical characteristics, microbiology and treatment outcomes were collected using a standardised form. The study also compared treatment costs between dalbavancin and hypothetical alternatives involving either inpatient care or OPAT. Clinical success was defined as the absence of definite failure based on the OVIVA (oral versus intravenous antibiotics) trial criteria. **Results**: a total of 39 patients were included, with a median age of 51 years (interquartile range (IQR) 40–72). Prosthetic joint infections (38 %) and septic arthritis (31 %) were the most common indications for dalbavancin use. The primary pathogens identified were *Staphylococcus aureus* (51 %) and coagulase-negative staphylococci (44 %). Dalbavancin was primarily chosen due to poor adherence or lack of OPAT options in 77 % of cases and for convenience in 23 %. In the necessity group, the use of dalbavancin resulted in a median cost saving of GBP 8894 per patient, and 31 inpatient days were avoided. Of the 32 patients (82 %) assigned a definite outcome, 72 % achieved clinical success. No significant adverse drug reactions were reported. **Conclusion**: this study fills an important evidence gap by demonstrating that dalbavancin is a viable and cost-effective option for MSKI patients that are unsuitable for oral or OPAT treatments. Dalbavancin facilitates early discharge, reduces hospital stays and achieves comparable clinical outcomes to conventional therapies.

## 1 Introduction

The treatment of complex musculoskeletal infection (MSKI) often requires surgery followed by prolonged antimicrobial therapy, which can delay patient discharge. Oral antibiotics are commonly used but are not always feasible due to resistance, intolerance or poor adherence (Azamgarhi et al., 2021). Outpatient Parenteral Antimicrobial Therapy (OPAT) is a potential alternative to improve quality of life, reduce the risk of hospital-acquired infections and lower costs (Chapman et al., 2019). However, not all patients are suitable for OPAT due to social circumstances, regimen complexity or the unavailability of OPAT services.

Dalbavancin is a lipoglycopeptide with high potency against a broad range of Gram-positive organisms. Its unique advantage is its extended half-life of 14.4 d, which avoids the need for daily dosing (Dorr et al., 2005). It is approved for treating acute bacterial skin and skin structure infection (ABSSSI). However, dalbavancin is increasingly used for off-label indications, including orthopaedic infections. Most evidence supports its effectiveness in the general treatment population rather than in specific cohorts where its unique properties could be most beneficial (Lovatti et al., 2023; Rappo et al., 2019).

Our study evaluates the effectiveness of dalbavancin in facilitating the early discharge of patients with MSKI who are not ideal candidates for oral or OPAT treatment. The specific objectives were to describe patient selection and assess the impact on healthcare service utilisation, clinical outcomes and treatment tolerability.

## 2 Methods

### 2.1 Study design and setting

This multi-centre retrospective cohort study was conducted across five hospitals in different regions of the United Kingdom and France between January 2017 and December 2022. The UK study sites included three large teaching hospitals in London, Southampton and Hampshire, as well as one specialist orthopaedic hospital in Stanmore. One further study site in France was a university hospital in Paris.

All adult patients aged 18 years or older who received dalbavancin to treat orthopaedic infections during the study period were included.

### 2.2 Patient population

Patients were eligible if they were aged ≥18 years and received dalbavancin for the treatment of MSKI, including septic arthritis, osteomyelitis, prosthetic joint infection (PJI) and other orthopaedic metalwork-related infections.

## 3 Data collection

Data were collected retrospectively using a standardised data extraction pro forma completed by the infection team using local infection registries at each study site. Information was obtained from electronic health records, including inpatient and outpatient notes, medication administration records, laboratory results, and microbiology reports.

Data collected included demographic characteristics, comorbidities (including the Charlson Comorbidity Index and baseline renal function) and treatment details, including indication for dalbavancin treatment, isolated pathogens, antimicrobial susceptibilities, dalbavancin dosing regimen, total duration of antibiotic therapy and concomitant antibiotics.

We recorded reasons why dalbavancin was chosen over alternatives. Local infection teams were asked for a preferred hypothetical alternative antimicrobial regimen if dalbavancin had not been used. Details of the comparator regimen included the specific antibiotic(s), dosing, duration and patient's suitability for OPAT.

## 4 Outcome measures

The primary outcome was healthcare utilisation, quantified by comparing treatment costs between dalbavancin and the patient-specific costs of administering theoretical alternatives via inpatient care or OPAT.

Drug costs were based on the British National Formulary. Dalbavancin treatment costs were based on the initial dose administered during an inpatient stay and subsequent doses administered to day attendees at the hospital infusion suite. Treatment costs for theoretical alternatives, including OPAT costs or inpatient care, were based on National Health Service (NHS) tariffs. OPAT costs included peripherally inserted central catheter (PICC) line insertion, maintenance, and healthcare professional visits for antibiotic administration and monitoring. For the French participating hospital, costs in euros were converted to pounds sterling using the average exchange rate for the study period.

Secondary outcomes included the incidence and type of adverse drug reactions, and clinical outcome was assessed at the last available follow-up. Definite failure, as defined by the OVIVA (oral versus intravenous antibiotics) study protocol, was defined as isolating indistinguishable bacteria from multiple samples, isolating a pathogenic organism from a single closed biopsy, positive diagnostic histologic findings, draining sinus tract formation or recurrence of frank pus adjacent to the affected area (Li et al., 2019). Patients who were neither readmitted to the initial hospital nor returned for follow-up evaluation were considered to have indeterminate outcomes. We considered treatment successful if the patient did not meet the criteria for definite failure at the last follow-up.

## 5 Subgroup analysis

Patients were divided into two subgroups based on the primary reason for choosing dalbavancin: (1) necessity for discharge, defined as cases where inpatient care would have been required to administer the alternative regimen due to lack of viable outpatient oral or OPAT options (e.g. poor adherence, social circumstances or complexity of alternative intravenous regimens), and (2) convenience, defined as cases where dalbavancin was preferred to avoid the need for an indwelling intravenous line and daily administration of OPAT.

### 5.1 Statistical analysis

Descriptive statistics were used to summarise baseline patient characteristics. Continuous variables were reported as means and standard deviations or medians and interquartile ranges (IQRs), as appropriate. Categorical variables were summarised as frequencies and percentages. Cost data were reported as medians and IQR per patient due to the anticipated non-normal distribution. The cost difference between dalbavancin and the theoretical alternative was calculated by subtracting the total cost of dalbavancin treatment from the estimated costs of the alternative regimen. Costs were reported separately for the necessity and convenience subgroups.

All data analyses were performed using SPSS version 26.0 (IBM Corp., Armonk, NY, USA).

## 6 Results

Thirty-nine patients were recruited from five sites: Southampton (14), Hampshire (10), Stanmore (8), London (2) and Paris (5). Twenty patients (51 %) were male with a median age of 51 years (IQR 40–72 years). The baseline demographic and clinical characteristics are summarised in Table 1. The median Charlson Comorbidity Index was 3 (IQR 1–5). Indications for dalbavancin treatment were evenly distributed between native bone and joint infections ( n=18 , 46 %) and implant-related infections ( n=21 , 54 %), with PJIs being the most common implant infection ( n=15 , 38 %).

**Table 1:** Baseline characteristics, reasons for dalbavancin use and sequential therapy timing.

Variable	N=39
	N (%)
Age (years), median (IQR)	51 (IQR 40–72)
Male sex	20 (51)
Charlson Comorbidity Index, median (IQR)	3 (1–5)
Indication	
Metalwork-related infection	
Prosthetic joint infection	15 (38)
Vertebral (metal)	3 (8)
Other orthopaedic metalwork infection	3 (8)
Native	
Chronic osteomyelitis	1 (3)
Vertebral osteomyelitis	5 (13)
Septic arthritis	12 (31)
Organism(s) identified	
*Staphylococcus aureus*	20 (51)
Methicillin-resistant	5 (13)
Coagulase-negative staphylococci	17 (44)
Polymicrobial infections	5 (13)
No cultured organism	2 (5)
Reasons for dalbavancin use	
Necessity	30 (77)
Poor adherence	24 (62)
Lack of OPAT options	6 (15)
Convenience	
To avoid OPAT	9 (23)
Days of antibiotics before first dalbavancin dose, median (IQR)	12 (IQR 6–17)
Total intended duration of dalbavancin, median (IQR)	34 d (IQR 21–50)
Theoretical alternatives	
Necessity	
Flucloxacillin	9 (23 %)
Vancomycin	7 (18 %)
Daptomycin	5 (13 %)
Teicoplanin	5 (13 %)
Linezolid	2 (5 %)
Doxycycline	1 (3 %)
Tedizolid	1 (3 %)
Convenience	
Daptomycin	5 (13 %)
Teicoplanin	2 (5 %)
Ceftriaxone	2 (5 %)

Staphylococcus species were the predominant pathogens, with *Staphylococcus aureus* isolated in 20 cases (51 %) and coagulase-negative staphylococci in 17 cases (44 %). Five cases (13 %) were polymicrobial infections involving Gram-negative pathogens, and two cases (5 %) were culture negative.

Fifteen patients (38 %) received concurrent antimicrobial therapy alongside dalbavancin. Among these, four were administered ciprofloxacin for associated Gram-negative infections, two received doxycycline (both continued as follow-on treatment) and one was given Co-trimoxazole for a culture-negative infection. Seven patients were treated with concurrent rifampicin, and one patient received quadruple therapy for tuberculosis.

### 6.1 Reasons for dalbavancin use and treatment details

The reasons for choosing dalbavancin were necessity in 30 cases (77 %) and convenience in 9 cases (23 %) (Table 1). Among the necessity group, poor adherence was the primary reason in 24 patients (62 %), all of whom were people who inject drugs (PWID). Six patients (15 %) lacked viable OPAT options due to multiple allergies, intolerance or antimicrobial resistance.

Dalbavancin was used as the primary treatment in 12 patients (31 %) and sequentially used in 27 patients (69 %) after a median of 12 d (IQR 6–17 d) of initial antibiotic therapy. The median intended duration of dalbavancin treatment was 34 d (IQR 21–50 d).

**Figure 1:**
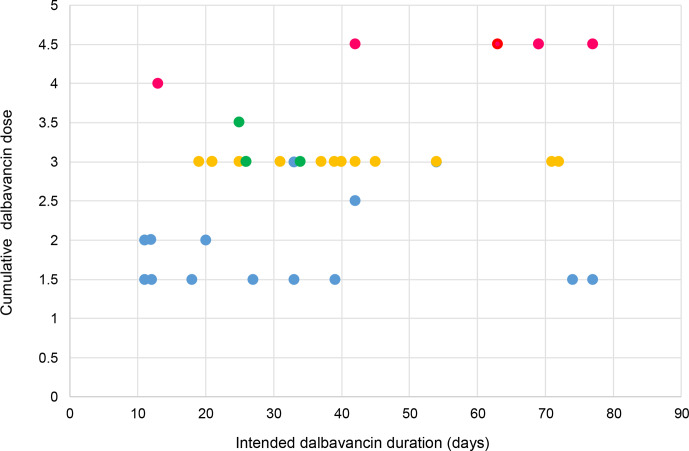
A scatter plot illustrating the cumulative dosing regimens administered by the intended treatment duration of dalbavancin. Colours correspond to the number of intended administrations (blue = 1,orange = 2, green = 3 and pink = 4). ^*^ Note that 12.9 % (4 of 31) of patients requiring repeat dosing did not attend their follow-up dose.

Several dosing regimens were used, as illustrated in Fig. 1. Thirty-one patients required outpatient re-dosing, though four missed their scheduled re-dosing. All of these were in the necessity group. The most common cumulative doses were 1.5 and 3 g, which covered treatment durations ranging from 10 to 75 d. The most frequently used regimen was a cumulative dose of 3 g administered as two 1.5 g doses 1 week apart ( n=20 , 51 %). A weak positive correlation was observed between the cumulative dalbavancin dose and the intended treatment duration ( $R^{2}=0.1996$ ).

### 6.2 Healthcare utilisation and costs

The cost analysis results are presented in Table 2. There were substantial differences in costs depending on whether dalbavancin was used for necessity or convenience. The median drug costs for the theoretical alternatives were higher for convenience, i.e. GBP 3120 per patient (IQR 1039–5400), than for necessity, i.e. GBP 600 per patient (IQR 461–1565). However, the treatment costs of administering the theoretical alternatives were significantly higher for necessity, i.e. GBP 10 083 per patient (IQR 7812–15 910), compared to convenience, i.e. GBP 3070 per patient (IQR 2908–3718).

**Table 2:** Cost analysis according to the reason for using dalbavancin.

Cost	Group	Necessity	Convenience
category		median	median
		(IQR) cost	(IQR) cost
		in GBP	in GBP
Dalbavancin	Drug costs^a^	1676	3352
		(1676–3351)	(3352–3352)
	Healthcare administration	461	461
	costs^b^	(461–921)	(461–461)
	Total costs	2366	3813
	(drug + healthcare costs)	(2137–3961)	(3813–4161)
Theoretical	Drug costs^a^	600	3120
alternative		(461–1179)	(1039–5400)
	Healthcare administration	9758	3070
	costs^b^	(6963–14 083)	(2908–3718)
	Total costs	10 883	6437
	(drug + healthcare costs)	(7812–15 910)	(5326–7811)
Relative cost saving of dalbavancin		8894	2445
versus alternative (theoretical – dalbavancin)		(5010–12 514)	(1053–4300)

^a^ Drug costs are based on the British National Formulary, accessed on 28 May 2024. ^b^ Hospitalisation costs are based on the National Health Service tariffs.

For the necessity group, the use of dalbavancin resulted in a median cost saving of GBP 8894 (5010–12 514) per patient, and a median of 31 inpatient days were avoided, which would have been required for the theoretical alternatives. For the convenience group, the median cost difference was GBP 2445 (IQR 1053–4300) in favour of dalbavancin, reflecting the lower healthcare costs compared to OPAT alternatives. The number of bed days saved was a median of 31 d, whereas no days were saved when used for convenience.

## 7 Clinical outcomes and safety

Table 3 summarises the clinical outcomes. Overall, 9 out of 39 patients (23 %) met the criteria for definite treatment failure, while 23 (59 %) achieved success at 12 months (IQR 6–17). The remaining seven patients (18 %) did not return for follow-up and were considered to have indeterminate outcomes.

**Table 3:** Clinical outcomes of dalbavancin by subgroup.

Subgroup	Definite	Indeterminate	Success	Follow-up
	failure	n (%)	n (%)	in month
	n (%)			(median,
				IQR)
Necessity^*^	6 (20)	7 (23)	17 (57)	12 (7–25)
Convenience	3 (33)	n/a	6 (67)	9 (6–13)

^*^ The success rates are reported for necessity considering the high rate of indeterminate outcomes due to follow-up issues, providing two interpretations based on whether indeterminate cases are considered failures or potential successes. n/a means not applicable.

When assessing outcomes by subgroup, for the convenience group three out of nine patients (33 %) met the criteria for definite treatment failure, while the remaining six patients (67 %) achieved success. For the necessity group, 6 out of 30 patients (20 %) met the criteria for definite treatment failure, with 17 patients (57 %) achieving success. All seven patients (23 %) not returning for follow-up were in the necessity group and were considered as having indeterminate outcomes. All of these patients had a history of poor adherence, and none were readmitted to the initial hospital. Consequently, the proportion of patients achieving success ranged from 57 % (when considering indeterminate cases as failures) to 77 % (when considering indeterminate cases as potential successes) over a median follow-up period of 12 months (IQR 6–17).

No significant adverse drug reactions associated with dalbavancin were reported, including allergy or associated renal impairment.

## 8 Discussion

Recent trends suggest increasing off-label use of dalbavancin for treating orthopaedic infections. A recent systematic review and meta-analysis supports dalbavancin as being safe and comparably effective to conventional antibiotics, with the potential to reduce healthcare costs (Lovatti et al., 2023; Rappo et al., 2019).

Our study evaluates the benefits of dalbavancin in a challenging cohort of patients. Most of them were not ideal candidates for daily antibiotic therapy. These patients are underrepresented in registration trials, which typically focus on the broader orthopaedic infection population. At one study centre, we calculated that approximately 2.4 % of staphylococcal orthopaedic infections necessitated treatment due to concerns with non-adherence. Although uncommon, these situations can significantly impact quality of life and healthcare resources.

In our study, poor adherence was the most common reason for using dalbavancin, accounting for two-thirds of all patients. Drug use was the most common cause of poor adherence and posed significant therapeutic challenges. OPAT is a significant risk due to the potential for line misuse, and oral treatments also offer little guarantee of adherence. Even when considering inpatient treatment, early discharge rates are reported at 61 % among PWID, who require prolonged antibiotic therapy (Buehrle et al., 2017). These factors are likely to result in premature treatment discontinuation. Our data on overall adherence of 90 % for re-dosing indicate that most patients returned for subsequent doses. Nevertheless, dalbavancin's long half-life is particularly beneficial for this subgroup due the ability to administer higher cumulative doses early in the treatment course to ensure continued therapeutic effects.

In the convenience group, we found success rates 67 % at 9 months (IQR 6–13). In the necessity group, the success rates were lower, but that could be due to the higher proportion of indeterminate outcomes. This is a consistent challenge across similar studies and complicates the true evaluation of outcomes in this patient population. These rates are generally lower than the reported success rates of dalbavancin for orthopaedic infections in the general population (Lovatti et al., 2023). Reasons for this are multifactorial, including factors such as availability of source control, comorbidities and previous treatment failures, particularly among patients with limited options and poor adherence to biofilm-active antibiotic options. Therefore, these lower success rates cannot be solely attributed to the choice of antibiotic.

There were no adverse drug reactions (ADRs) to dalbavancin reported in our study. This is consistent with other studies of off-label use in orthopaedic infections that show it is generally safe and well tolerated in 90 % of patients, supporting its use in moderate-length treatment regimens (Lovatti et al., 2023).

Despite its benefits, the use of dalbavancin is complicated by the lack of readily available sensitivity testing, which often relies on inference from vancomycin susceptibility. Furthermore, well-defined dosing and monitoring guidelines are limited. Various regimens recommend administering doses ranging from once to twice a week, with specific loading and maintenance doses. Recent consensus is based on a fixed duration for the full treatment course (Senneville et al., 2023). A two-dose regimen of 1500 mg on days 1 and 8 provides sufficient distribution of the drug into bone and articular tissue to achieve tissue exposure over the dalbavancin minimum inhibitory concentration (MIC) for *Staphylococcus aureus* for 8 weeks (Dunne et al., 2015). This dosing regimen was found to be well tolerated and effective, with clinical cure of 97 % at 6 weeks, as well as 96 % at 6 months and 1 year (Rappo et al., 2019). This was the most used regimen in our study and has the advantage of convenience due to less frequent dosing. We found more variability around dosing for longer durations, and there may be a role for therapeutic drug monitoring (TDM). This approach may reduce the potential for sub-therapeutic concentrations, which is an important consideration for preventing the development of resistance, as emerging resistance has been reported during treatment with dalbavancin for PJI (Zhang et al., 2022). Additionally, TDM could contribute to cost savings by optimising dosing strategies. The authors would welcome the availability of dalbavancin susceptibility testing and TDM to enhance its clinical application further.

Using dalbavancin out of necessity resulted in GBP 8894 per patient cost avoidance, primarily due to costly extended inpatient stays. We also found that the cost difference for dalbavancin is smaller and in favour of using dalbavancin when used for convenience, which closely reflects the lower healthcare costs compared to alternative options in OPAT. However, whether there is a cost reduction with dalbavancin largely depends on the specific drug administered and whether the patient would have been suitable for self-administration, which can reduce treatment costs. We did not consider the costs of managing complications. Our findings demonstrate that the healthcare costs avoided due to dalbavancin often offset its higher acquisition costs, which can be a barrier to its use.

Our investigation acknowledges several limitations that warrant mentioning. Using theoretical comparators to evaluate dalbavancin impact was practical, as it required centres to provide hypothetical treatment regimens that mimic real-world settings. However, we assumed all patients with adherence issues would need inpatient care as the more reliable option than OPAT or oral therapies for ensuring adherence, even when it might not always be required. This assumption may bias the cost-effectiveness of dalbavancin.

Dalbavancin was predominantly used as sequential therapy; therefore, success following antimicrobial treatment cannot be attributed solely to dalbavancin. Another limitation is the frequency of indeterminate outcomes due to patient attrition, which, while difficult to prevent, potentially distorts the perceived efficacy of the treatment. Nevertheless, our study focuses on a cohort frequently excluded from clinical trials, who stand to benefit notably from dalbavancin's unique characteristics.

Our study's multi-centre design broadens the applicability of its findings, providing a more comprehensive view of dalbavancin's effectiveness and safety in this challenging cohort. This information is confirmatory and supplements the wider trial data to fully determine dalbavancin's utility.

## 9 Conclusion

Dalbavancin is a viable option in patients with MSKI where conventional antibiotic options are unsuitable. Its use was associated with comparable treatment outcomes to alternatives, with no patients experiencing adverse drug reactions. Dalbavancin significantly reduced overall treatment costs compared to an inpatient hospital stay.

## Data Availability

The datasets used and/or analysed during the current study are available from the corresponding author on reasonable request.
